# Supplementary material for: Effect of justification of wife-beating on experiences of intimate partner violence among men and women in Uganda: A propensity-score matched analysis of the 2016 Demographic Health Survey data
Source: PLoS One. 2023 Apr 12;18(4):e0276025. doi: 10.1371/journal.pone.0276025 (PMC10096235; doi:10.1371/journal.pone.0276025)
Supplement: Attachment — Submitted filename: Response to Reviewers_PONE-D-22-26659R1.docx [file pone.0276025.s001.docx]

| **Reviewer Comments** | **Response to Reviewer Comments** |
| --- | --- |
| Reviewer #1: |  |
| Abstract |  |
| 1. Why not you used structured abstract form (kindly write the parts of an abstract in a separate line). | We have used a structured abstract in the revised submission detailing the introduction, methods and materials, results, and conclusion. |
| 2. “…the majority (3,774; 78.3%) were women compared to men (1,047; 21.7%)” with regard to what, I think something is missed in this sentence. | The sentence has been revised for clarity as below  “Results showed that a total of 4,821 (46.5%) out of 10,394 respondents reported that a husband is justified in beating his wife for specific reasons. Among these the majority (3,774; 78.3%) were women compared to men (1,047; 21.7%).” |
| 3. “We found that at population level…” how do you address multilevel factors by using an ordinary logistic regression? | We appreciate this observation and have edited this sentence to read as below:  “Overall, we found that men and women who accept gender norms justifying wife-beating are more likely to experience all three forms of IPV” |
| 4. “Government should tackle the drivers of acceptability of gender norms justifying wife beating at the societal level.” What are the drivers of acceptability of gender norms justifying wife beating? Do you obtain those drivers in your study? Be specific with your recommendations? | This is another good observation because we did not tackle such drivers in our study. The DHS data doesn’t not explicitly have indicators for drivers of acceptability of gender norms justifying wife-beating. In line with our analytical approach, we have edited the sentence to read as below:  “There is, therefore, a need for more research to study drivers for acceptance of gender norms justifying wife-beating to enable appropriate government agencies to put in place mechanisms to address the acceptability of gender norms justifying wife-beating at the societal level” |
| Introduction |  |
| 5. Para 1, line 2-5 is too long. Please paraphrase it. | The sentence has been re-written an split into two |
| 6. Please try to show the problem, gaps and tried solutions to reduce IPV globally, in SSA,,,, Uganda. | We have re-written the introduction section to address this. We have also provided the needed references. |
| 7. Line 13-16 needs a reference.  8. Line 15-6, “several programmes aimed at shifting norms and behavior around IPV have been implemented across Africa” what types of programmes were implemented? What was their outcome? What type of norms and behaviors they intended to change? Please explain with examples. | We have re-written the introduction section to address this. We have also provided the needed references. |
| 9. Please write the long version of “SASA! “? | We have provided a general but short description of the SASA! Program. We have also provided 6 references that have a detailed description of the SASA! Program.  It is our considered view that it is not appropriate to replicate the detailed description of the SASA! Program in our paper as this is already explained elsewhere and the appropriate citations have been provided. |
| 10. Line 20, needs a reference.  11. Line 24 needs a reference.  12. Line 26 needs a reference. | We have re-written the introduction section provided the needed references. |
| Methods and Materials |  |
| 13. How do use quasi experimental design for secondary data? What does experimental study design mean? What is the difference between experimental and retrospective study designs? In quasi experimental design you are going to assign the subjects into control and study/intervention group then you will measure your outcome variable both at the base line, and end of your intervention, please make it clear in the methods part specifically in exposure part. | In simple terms, the model is premised on what would happen to a group of targeted individuals if they had no access to the intervention (in our study). In the absence of any experimental data, the unobserved outcome for each individual in a cross sectional study is constructed using propensity score matching. The Propensity Scores Matching (PSM) is the probability of assignment to a particular intervention conditional on observed covariates.  Technically, unlike a randomized controlled trial (RCT), quasi-experimental studies do not randomize participants to intervention or control arms but rely on natural exposures. We agree that quasi-experimental studies sometimes allow follow-up to assess outcomes following and exposure. However, in the present study, there is no follow-up because we used existing data, the UDHS 2016 data, to conduct causal-effect analysis. Basically, we compared outcomes between the exposed (participants justifying wife beating) and the unexposed groups (participants not justifying wife beating) after balancing observed characteristics. To do so, we used Propensity Scores Matching (PSM).  The rationale for use of the PSM approach and data analysis technique are well described in the respective sections within the manuscript. |
| 14. What does mean gender norm? What type of norms (positive, negative) are you going to assess as a means of justification? | Gender norm is a social construction of expected roles or behavior for men, women, girls and boys in society. Harmful (negative) gender norms are those that affect the wellbeing of an individual for instance justification of violence would be considered as negative. Positive (or progressive) norms are those that address inequalities between men and women and enable them perform their roles in a nondiscriminatory way for instance norms supporting equal access to resources, education among others. See e.g., WHO (n.d). Gender and Health. Available at <https://www.who.int/health-topics/gender#tab=tab_1> |
| 15. Put references to your outcome variables (line 104-112) | We had already mentioned that our study is based on the 2016 UDHS and provided the citation in the section “**Data Source**”. To provide more clarity, we have included the same reference – the 2016 UDHS – in this section. |
| 16. Line 114-124, where and how do you get the independent variables? | We had already mentioned that our study is based on the 2016 UDHS and provided the citation in the section “**Data Source**”. To provide more clarity, we have included the same reference – the 2016 UDHS – in this section. |
| 17. Why you measure life time prevalence rather 12months prevalence of IPV? Which one do you think more important for public health intervention? | It is well documented that any exposure to violence contributes to the genesis of, and exacerbates, mental health conditions, and existing mental health problems increase vulnerability to partner violence, and creates a loop that imprisons victims and perpetuates the abuse in intimate relationships e.g see Mazza M, Marano et al (2021). Intimate partner violence: A loop of abuse, depression and victimization. World J Psychiatry;11(6):215-221. doi: 10.5498/wjp.v11.i6.215.  We considered violence committed by the current spouse/partner (for currently married women and men) and by the most recent spouse/partner (for formerly married women and men). As such, it is our considered view that any act of gender-based violence that results in, or is likely to result in, physical, sexual, or mental harm or suffering, is of major public health concern – whether it occurred in the past 12 months or happened several months ago. |
| 18. Why do not you compute the composite variable of IPV? | The focus of the current paper is in examining the distribution of respondents’ experiences of different forms of IPV (outcomes) by the justification of wife-beating (exposure) as shown in Tables 3 and 4.  It is our considered view that experiences of different forms of IPV are neither additive nor multiplicative. In one of our other papers, we have shown that coexistence of different forms IPV is common at country level. See Kiwuwa-Muyingo S, Kadengye DT (2020). Prevalence and Risk Factors for Women’s Reports of Past-Year Intimate Partner Violence: A Comparative Analysis of Six East African National Surveys. J Interpers Violence. Doi:10.1177/0886260520969374  Moreover, the DHS tool is set up in such away that the 3 forms of IPV are assessed on independent scales, that is, using different sets of indicators. Therefore while the idea is good, we believe that a composite score would require a tool specifically designed to address the composite abusive scale  Of course, we could have computed a variable on “experiencing *any* of the three forms of IPV”. However, we would have lost information on the variability of estimates for the different forms of IPV, which we believe may be used to guide programmatic interventions. |
| 19. How do you pair each pre-test score to the corresponding post-test score? How do you obtain your post-intervention/test score? How do you prefer retrospective cohort over quasi experimental design for your study? You need to clarify it | Our study is not a pre and post-test or a retrospective cohort study. This is a quasi-experimental study that compared 2 groups of participants: a group justifying wife-beating (exposed group) and another group that never justified wife beating (unexposed group). To estimate causal effect, we made the groups balanced on observed characteristics using PSM approach. Now, once you perform PSM, participants in the exposed group get matched with those in the unexposed group on similar propensity scores while others with different propensity scores do not. The statistical software thus automatically generates the matched pairs. The unmatched are then dropped from the analysis. Our data analysis section has elaborated on this in-depth. |
| 20. How do you control the effect of other covariates on IPV? How do you know the real effect of your independent variable to the outcome variable? | We did not measure the effect of other covariates on IPV. We measured the effect of gender norms justifying wife beating (exposure) on experiences of IPV (the outcome). To do so, we fitted a regression model of the exposure on the outcome while removing the effect of observed covariates by balancing all of them across the exposed versus unexposed groups using PSM. This way, the groups became the same on the measured covariates. Once the covariates are balanced, the question for PSM approach is then the impact of the intervention conditional on covariates. This is the basic idea of matching, that is, to find in a group of participants (without the intervention), the individuals who are similar to those with the intervention at baseline.  We acknowledge that PSM balances measured covariates so we had acknowledged its limitation regarding unmeasured confounders in our earlier submission.  In our revision manuscript, we modified the earlier sentence to reads:  "Some limitations in this study include reliance of the analytic technique on observed covariates but not unobserved (unmeasured) covariates". |
| 21. Why you are interested to study the effect of acceptability of gender norms justifying wife-beating on the life-time experiences of sexual, emotional, or physical intimate partner violence (IPV)? Why not other variables? Why you are interested to study among the two groups (men and women)? | The reasons behind this interest has been further clarified in the Introduction Section of the revised paper see paragraphs 3 – 4. Specifically, in our paper, it is stated thus:  **Para 3:**  “Strong evidence exists that social norms about the roles and behavior of men and women contribute to an increased level of IPV in various low- and middle-income countries [3,4,8,9]. Given the importance of social norms such as male masculinity and female subordination in shaping acceptable behavior within communities, several programmes aimed at shifting norms and behavior around IPV have been implemented across Africa [10]. For example, the SASA! Project, a community mobilization project designed to transform gender relations and power dynamics, and reduce the social acceptability of IPV has been implemented in over 15 countries, including Uganda [10–14]. The general strategy of the SASA! Project and several of its variants is to bring about changes in social norms through local activism, media, use of communication materials and community-based edutainment/theatre, and advocacy in order to reduce the social acceptability of IPV [10,14].”  **Para 4:**  “………This could be a consequence of women’s perceived low status in many societies and men’s dominance over women which is enforced through sexual and physical violence [19]. The close association of violence with masculinity has created a dangerous and unjust power dynamic that manifests in forms of violent physical, emotional, or psychological aggression and affects an alarming proportion of the population [2]. In some communities rationalization of men’s controlling attitudes is associated with the justification of gender norms such as wife-beating [20], which may increase the risk of experiencing IPV [21,22].”  The effects of acceptability of gender norms justifying wife beating vary between women and men based on anecdotal data so uncovering these effects through a rigorous study is important. It is for this reason that we performed sub-group analysis for men and women and as you can see from the results, the effects are different. |
| Conclusions and Discussions |  |
| 22. Please mention them separately. Your conclusion should follow the discussion. | In terms of the structure, we followed the Author Guidelines for PLOS available at <https://journals.plos.org/plosone/s/submission-guidelines>  Specifically, it is stated under **“Results, Discussion, Conclusions”** that ‘these sections may all be separate, or may be combined to create a mixed Results/Discussion section (commonly labeled “Results and Discussion”) or a mixed Discussion/Conclusions section (commonly labeled “Discussion”)’.  In our earlier submission, we used the mixed Discussion/Conclusion option as guided by PLOS ONE guideline. However, we have now separated the two sections per this guidance. |
| 23. Your discussion seems result. It needs further clarification and comparison with other studies. Please rewrite and try to widen your discussion according to your findings. | Thank you for this. We have enhanced this section and provided comparison with additional studies in addition to what we had initially included. |
| 24. Line 230 “Gender-based violence (GBV) of any form perpetrated against a woman or girl, man or 231 boy harms their physical, sexual or psychological health”. Why you mentioned GBV in this section? Do you think IPV and GBV are similar? | This is a good observation. IPV is not necessarily similar to GBV.  IPV specifically describes violence that occurs between people in sexual or romantic relationships and can occur between partners of the same gender identity. On the other hand, GBV is directed against a person because of their gender and can include IPV, sexual violence by perpetrators other than an intimate partner, child marriage, and female genital mutilation, among others.  This part has been edited. |
| 25. “In this paper, we investigated the causal effects of acceptability of gender norms justifying wife-beating on the life-time experiences of sexual, emotional, or physical intimate partner violence (IPV) among Ugandan men and women using a quasi-experimental design with propensity-score matched (PSM) analysis. We analyzed 235 data from the 2016 Uganda Demographic and Health Survey (UDHS) data” this sentence should come at the beginning of your discussion, so avid the sentence about GBV preceding it. Then rewrite it as “the aim of this study was to investigate the causal effects of acceptability of gender norms justifying wife-beating on the life-time experiences of sexual, emotional, or physical intimate partner violence (IPV) among Ugandan men and women using 2016 Uganda Demographic and Health Survey (UDHS) data.” | This has been edited as advised. |
| 26. “, the exposure and outcomes were assessed by self-reports implying that the possibility of social desirability bias cannot be excluded” what was your effort to reduce this bias? | We did acknowledge that self-report is associated with social desirability and there was no effort to reduce this mainly because of the reasons given in the manuscript. Specifically, a key strength of our study is that we analyzed a large and nationally representative dataset, implying that the findings are generalizable to the entire country and other similar settings. Secondly, we applied a robust study design and analytic approach to measure unbiased cause-effect relationships using observational data, implying that results are robust to unmeasured confounders and the analytic technique |
| 27. “In conclusion, findings from this study show that the risk of experiencing IPV is high…..” how is your finding high? Compared to what? | This has been edited to read as: “In conclusion, findings from this study show that the likelihood of experiencing sexual, emotional, and physical IPV is higher among both women and men who accept gender norms justifying wife-beating compared to those who do not.” |
| 28. “…..There is, therefore, a need to tackle the drivers of gender norms justifying wife-beating at the societal level.” What are the driving factors? Your recommendation seems shallow please rewrite it and be specific with your finding? (What to do, how to do, who should do it)? | This has been edited to read:  “………There is, therefore, a need for more research to study drivers for acceptance of gender norms justifying wife-beating. In this way, appropriate government agencies can be able put in place mechanisms to address the acceptability of gender norms justifying wife-beating at the societal level. For instance, it has been recommended elsewhere that strategies for reducing gendered social norms related IPV could incorporate a wider range of individual, household, community, institutional, and societal factors that view violence in the family as unacceptable and that can be acted on in order to contribute to generational efforts to eliminate IPV [3]. One strategy could be for policy makers to repackage scientifically proven interventions such as the SASA! Program [11,12] with a combination structured legal platforms for perpetrators, as well as intensive programs to educate men, women, boys and girls to adopt more prohibitive social norms against negative attitudes such as justification of wife-beating for any reason.” |
| 29. Do you think you answered your research question? What was your hypothesis? | The aim of this study was to investigate the causal effects of acceptability of gender norms justifying wife-beating on the life-time experiences of sexual, emotional, or physical IPV among Ugandan men and women using a quasi-experimental design with propensity-score matched (PSM) analysis.  This objective has been addressed based on findings presented in Table 4. |
| 30. You have to acknowledge your study participants. | We did not interact with any study participants. As stated in the Ethical Statement, we stated that “We conducted secondary data analysis using the 2016 Uganda Demographic Health Survey de-identified data that is publicly available and requires no ethical approvals. However, permission to use these data was obtained from the Demographic and Health Surveys division at ICF International through completion of an online request form that is available on the DHS website (<http://www.dhsprogram.com>) from where the data was downloaded”.  This permission has been acknowledged in our acknowledgement section. |
| General comments |  |
| 31. How long do you follow your study subjects to measure the post-test score? What is your level of blinding? | We have read the comments #31-36 with huge interest and we thank you for raising them. Once again, we are happy to highlight that we analyzed existing observational data so there was no follow-up of any nature since this was not a randomized control trial or prospective cohort study. By default there is no blinding. Under the sub-heading "Exposure" in the methods section, we have clearly described the exposure and unexposed groups and it reads:  "The exposure of interest was acceptance of gender norms justifying wife-beating, with the exposed group being respondents reporting that it is acceptable and the non-exposed group consisted of respondents that reported it is not acceptable. During the 2016 UDHS, both women and men were asked to report whether or not beating one’s wife was justified under the following five circumstances: 1) Wife goes out without telling husband; 2) Wife neglects the children; 3) Wife argues with husband; 4) Wife refuses to have sex with husband; and, 5) Wife burns the food. The justification of wife-beating was computed as the percentage of all women and men aged 15-49 who agree that a husband is justified in beating his wife under the above five circumstances."  Synonymous to baseline data are the covariates considered in the study that preceded the outcome of interest. |
| 32. Please put a detailed description about your intervention? |  |
| 33. Intervention and control group? |  |
| 34. Outcome and exposure ascertainment issue is questionable? |  |
| 35. Baseline data, and intervention data is needed. |  |
| 36. The average or percentage of difference across groups in terms of outcome and exposure? |  |
|  |  |
| Reviewer #2: Comments |  |
| This paper makes important contribution to the literature on intimate partner violence in Uganda. Just a minor suggestion is that it should reflect the timeline of study. For example, A propensity matched scores approach on 2016 UDHS or something like that. Another thing is that it is vague whether the study was carried out among both married and never-married people. If not among never-married people, the title should be specified accordingly. For example, married men and women or couples. | This is a good suggestion and we have edited the title as such, much as this has come with the added cost of having a very long title of 27 words. The title now reads as:  “Effect of justification of wife-beating on experiences of intimate-partner violence among men and women in Uganda: A propensity matched scores analysis of the 2016 Demographic Health Survey Data”  This also is a good observation. As described in the methods section under the sub-title “Outcomes”, we considered all men and women in current or most recent intimate relationships (currently married, in intimate partner relationships, or most recent past marriage or intimate partner relationship). We are of the view that including this in the title will make it very lengthy. Moreover, the title already has the word “intimate-partner”. |
| Introduction |  |
| This session highlighted the linkage between justification of gender norms and the risk of IPV. One unclear thing is that the concept between justification of wife beating and the risk of IPV in men. Does it mean that a man who justified wife-beating has more experienced with IPV by his wife? Generally, justification of wife-beating is based on gender-norms that men should practice punishment acts on their wives. So, if a man justifies wife-beating, he will probably more perform IPV on his wife. Or does this area remain as a knowledge gap? | Thank you for raising this very important concern.  The introduction clearly describes the prevalence of IPV among men and women from the DHS data.  Wife beating in the context of DHS focuses on reporting whether or not beating one’s wife was justified under the following five circumstances: 1) Wife goes out without telling husband; 2) Wife neglects the children; 3) Wife argues with husband; 4) Wife refuses to have sex with husband; and, 5) Wife burns the food. There is social acceptance of male dominance in the hierarchy of power which justifies a man’s right to punish a woman who transgresses from traditional gender roles. The existing gender power imbalance embedded in patriarchy and women’s subordination regard to men’s power over women. While both men and women have the ability to be violent in relationships, it remains unclear to what extent women’s violence can be compared to men’s as perpetrator of IPV in the context and motivation for this.  Regarding the question on whether justification of wife beating increases the risk for IPV in men or whether justification of wife beating is related to previous experience of IPV from the wife, we do have data to support this argument at the moment. It is indeed a knowledge gap that could be explored for future studies within the paragraph on study limitations. |
| Research aim: The research aim well reflects the purpose of this study. | Thank you |
| Methodology |  |
| The use of propensity-score matched (PSM) analysis on a cross-sectional data is appropriate for this study that investigates causal effects. The sampling method is clear. However, is there any inclusion criteria or exclusion criteria for asking the question – justification of wife-beating? Was this question asked to every participant even if the participant was never married one? Then the responses were included in the analysis. If so, how it linked with IPV? According to Line 102-103, “[IPV] was measured by asking all ever-married women and men if their spouse/partner ever did the following to them”. | As explained above, we considered all men and women in current or most recent intimate relationships (currently married, in intimate partner relationships, or most recent past marriage or intimate partner relationship). To ensure this clarity, this section has been edited to read:  “ More specifically, violence committed by the current spouse/partner (for women and men currently married or in intimate relationships) and by the most recent spouse/partner (for women and men formerly married or in past intimate relationships) was measured by asking all women and men if their spouse/partner ever did the following to them:” |
| Regarding the covariates, does the partner’s alcohol consumption not have effect on experience of IPV? | It is well known that a partner’s alcohol consumption has an effect on experience of IPV. The UDHS 2016 report, page 321, states that:  “Women whose husband/partner is often drunk are much more likely (84%) to experience spousal violence than women whose husband/partner is sometimes drunk (66%), drinks alcohol but is never drunk (42%), or does not drink alcohol (45%). [Similarly] the wife’s/partner’s consumption of alcohol affects men’s experience of spousal violence in the same way as the husband’s/partner’s consumption affects women’s experience of violence” Ref: Uganda Bureau of Statistics (UBOS), ICF International. Uganda Demographic and Health Survey 2016 [Internet]. Kampala, Uganda and Rockville, Maryland, USA: UBOS and ICF; 2018. Available from: <http://dhsprogram.com/pubs/pdf/FR333/FR333.pdf>.  Therefore, alcohol could show a true cause-effect relationship in that alcohol may lead to IPV. We could use alcohol if we had evidence that alcohol consumption preceded occurrence of IPV. On a different front, alcohol consumption could show a reverse cause-effect relationship in that IPV makes people to drink alcohol. If we had this evidence, we would exclude alcohol if it never preceded IPV. But alcohol could be a confounder, in which case we would include it in the analysis.  Having said that, by its very nature, DHS includes many variables most of which were not included in our study, and it is our considered view that its inclusion of exclusion would not change the findings and/or conclusions of our results. After all, as indicated in the paper, the sensitivity analysis showed that the results are robust to unmeasured to unmeasured confounders. |
| Statistical Method |  |
| The use of STATA software is appropriate for this study. Detail explanation helped the reader in understanding the statistical procedure. | Thank you |
| Results |  |
| The finding represents the statistical analysis performed by the authors. | Thank you |
| Discussion |  |
| This study well discussed about the findings by using comparative global studies and sound theoretical background. Just a curiosity, why did the analysis not include the childhood experience of gender-based violence (e.g. Witnessing parental violence) as a covariate? | Whereas by design the UDHS 2016 collected information on many variables, the Domestic Violence Module did not ask respondents about their childhood experiences of GBV. Ref: Uganda Bureau of Statistics (UBOS), ICF International. Uganda Demographic and Health Survey 2016 [Internet]. Kampala, Uganda and Rockville, Maryland, USA: UBOS and ICF; 2018. Available from: http://dhsprogram.com/pubs/pdf/FR333/FR333.pdf |
| Conclusion |  |
| The authors’ recommendations are appropriate, but it would be more effective if the conclusion session is separately written from discussion session.  If available, it should suggest some interventions that could reduce gender norms justifying wife beating at the societal level.  Again, the concept for the increased risk of IPV in men who accepts wife-beating norms is still unclear. If this study does not explain, it should be suggested for future studies. | In terms of the structure, we followed the Author Guidelines for PLOS that ‘these sections may all be separate, or may be combined to create a mixed Results/Discussion section (commonly labeled “Results and Discussion”) or a mixed Discussion/Conclusions section (commonly labeled “Discussion”)’.  However, we have now separated the two sections per this guidance.  The conclusion section has been revised and now states as below:  “In conclusion, findings from this study show that the likelihood of experiencing sexual, emotional, and physical IPV is higher among both women and men who accept gender norms justifying wife-beating compared to those who do not. There is, therefore, a need for government to put in place mechanisms to address the acceptability of gender norms justifying wife-beating at the societal level. For instance, it has been recommended elsewhere that strategies for reducing gendered social norms related IPV could incorporate a wider range of individual, household, community, institutional, and societal factors that view violence in the family as unacceptable and that can be acted on in order to contribute to generational efforts to eliminate IPV [3]. One strategy could be for policy makers to repackage scientifically proven interventions such as the SASA! Program [11,12] with a combination structured legal platforms for perpetrators, as well as intensive programs to educate men, women, boys and girls to adopt more prohibitive social norms against negative attitudes such as justification of wife-beating for any reason.”  This is related to the previous comment and our response regarding the question on whether justification of wife beating increases the risk for IPV in men or whether justification of wife beating is related to previous experience of IPV from the wife. We do have data to support this argument at the moment. It is indeed a knowledge gap that could be explored for other studies. As advised, we have included this as a limitation and suggestion. |
| Grammatical errors |  |
| Please review the manuscript for grammatical and spelling errors. Minor error – [Line 240] Please revise the phrase (double justify words): “who did not justify justifying wife-beating” | We have proofread the manuscript for grammatical and spelling errors. |
